# Supplementary material for: Breath Ammonia Is a Useful Biomarker Predicting Kidney Function in Chronic Kidney Disease Patients
Source: Biomedicines. 2020 Oct 31;8(11):468. doi: 10.3390/biomedicines8110468 (PMC7692127; doi:10.3390/biomedicines8110468)

Supplemental Material

- **Figure S1.** Correlation between salivary pH value and blood urea nitrogen, serum creatinine, and estimated glomerular filtration rate. (A) Correlation between salivary pH value and blood urea nitrogen (BUN) level. (B) Correlation salivary pH value and serum creatinine level. (C) Correlation between salivary pH value and estimated glomerular filtration rate (eGFR).

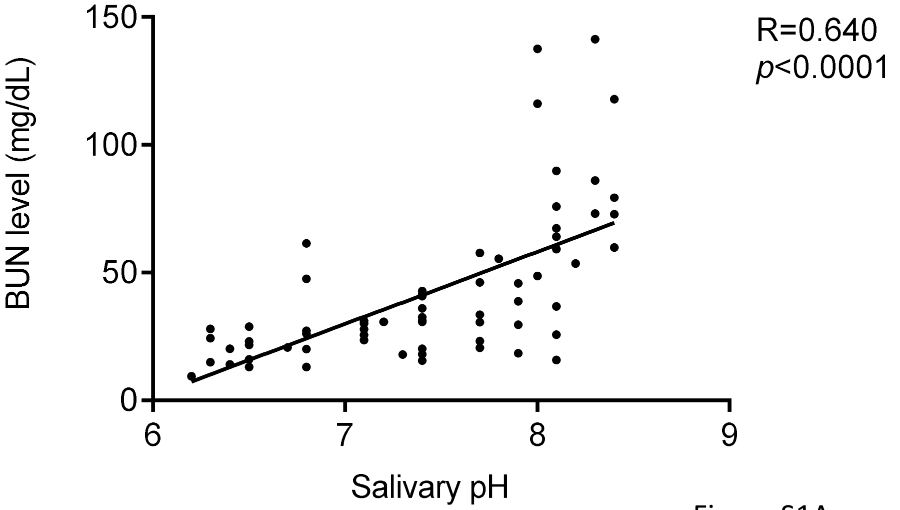

Figure S1A

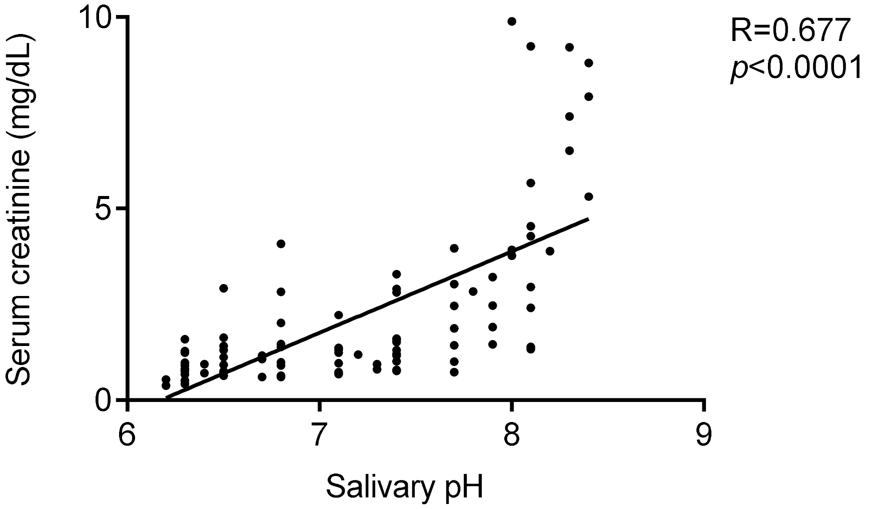

Figure S1B

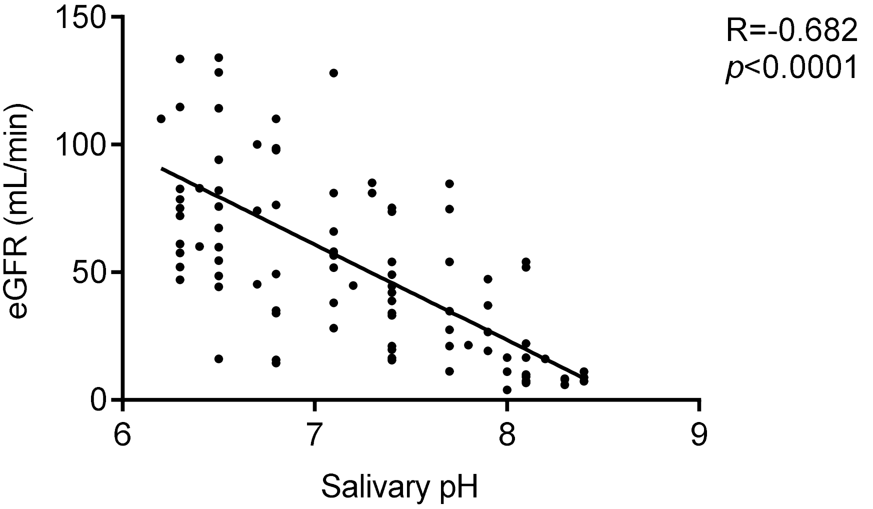

Figure S1C

Figure S2. Salivary pH value in patients with chronic kidney disease stage 1–5. Salivary pH values were measured in chronic kidney disease (CKD) stage 1–5 patients. Box plot is used to display salivary pH value in different CKD group, and significant differences between groups are indicated (*, p<0.05; **, p<0.01; ***, p<0.001; ****, p<0.0001).

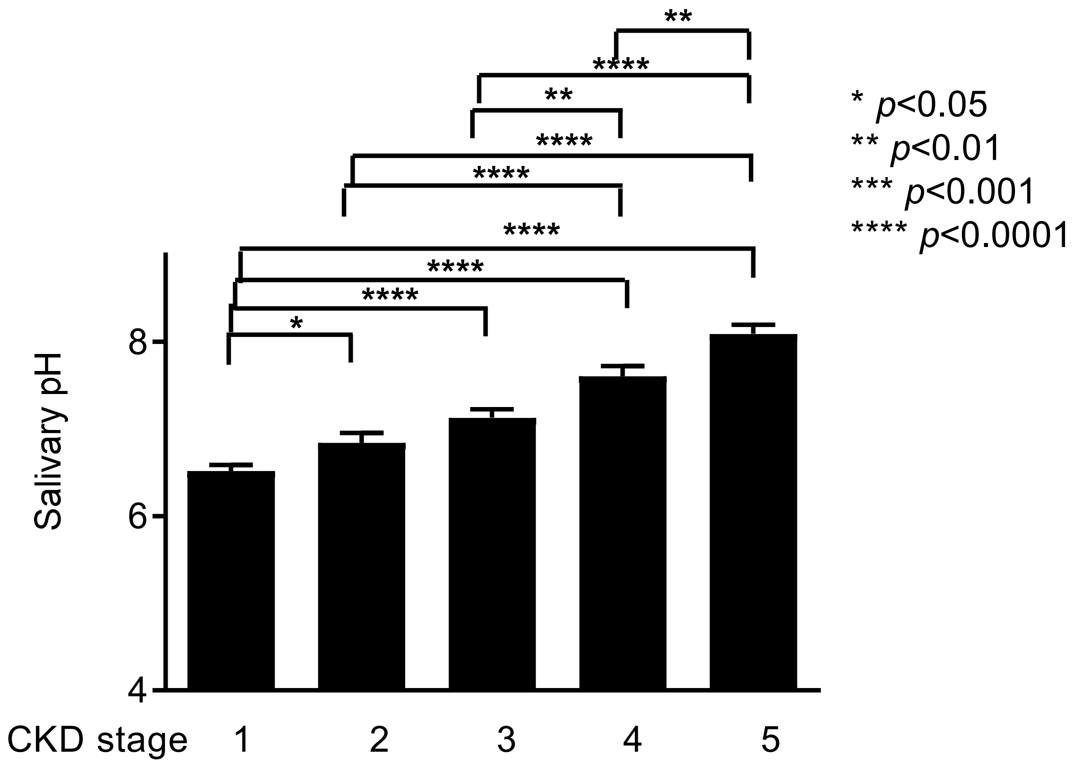

Figure S3. Correlation between breath ammonia concentration and age.

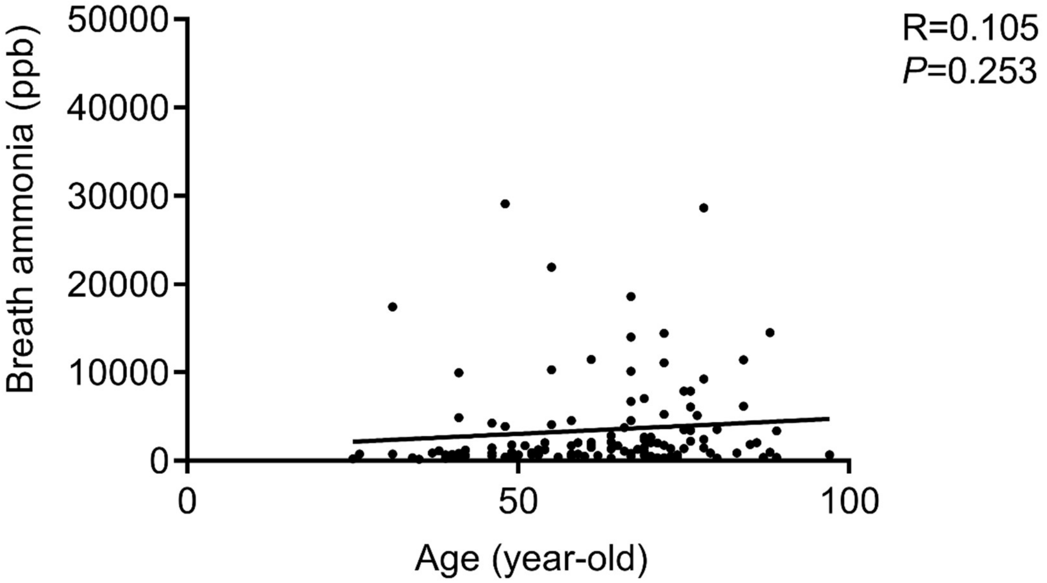

Figure S4. Receiver operating characteristic curve analysis of breath ammonia for screening patients with eGFR <60 mL/min/1.73 m2 derived from the combination of 121 patients from this study with previous 34 patients with chronic kidney disease stage 3–5.

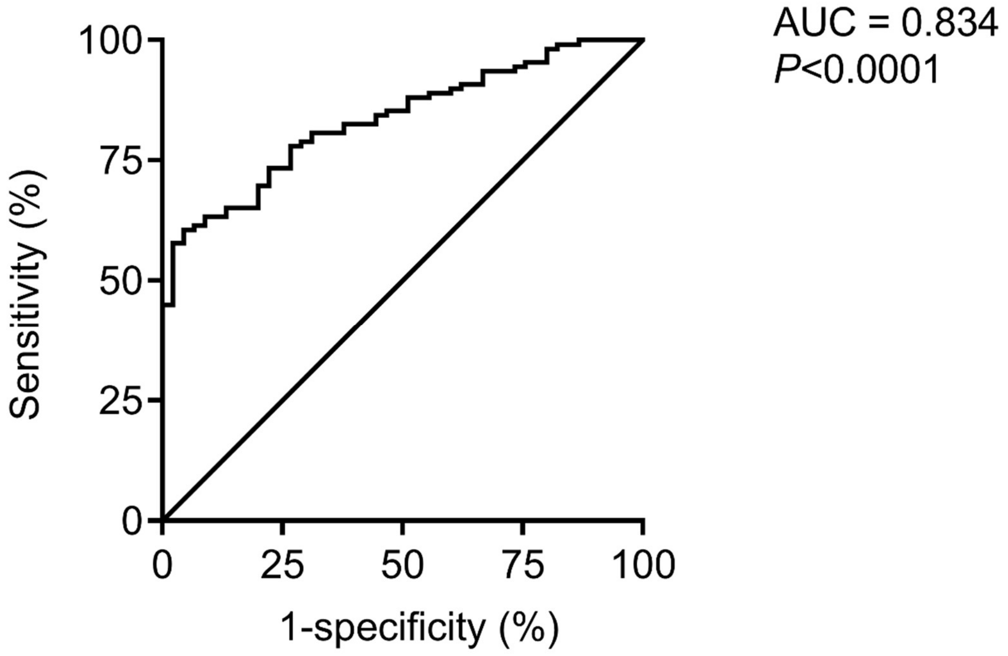

Supplement: Supplementary file 1 [file biomedicines-08-00468-s001.pdf]
